# Nine-year distribution pattern of hepatitis C virus (HCV) genotypes in Southern Italy

**DOI:** 10.1371/journal.pone.0212033

**Published:** 2019-02-20

**Authors:** Arnolfo Petruzziello, Rocco Sabatino, Giovanna Loquercio, Annunziata Guzzo, Lucia Di Capua, Francesco Labonia, Anna Cozzolino, Rosa Azzaro, Gerardo Botti

**Affiliations:** 1 SSD Virology and Molecular Biology, Department of Diagnostic Area, Istituto Nazionale Tumori, Fondazione “G. Pascale”, IRCCS Italia, Naples, Italy; 2 Transfusion Service, Department of Hemathology, Istituto Nazionale Tumori—Fondazione “G. Pascale”, IRCCS Italia, Naples, Italy; Centers for Disease Control and Prevention, UNITED STATES

## Abstract

**Introduction:**

It has been greatly described that different hepatitis C virus (HCV) genotypes are strictly correlated to various evolution, prognosis and response to therapy during the chronic liver disease. Aim of this study was to outline the changes in the epidemiology of Hepatitis C genotypes in Southern Italy regions from 2006 to 2014.

**Material/Methods:**

Prevalence of HCV genotypes was analyzed in 535 HCV-RNA positive patients with chronic Hepatitis C infection, selected during the period 2012–2014, and compared with our previous data, referred to periods 2006–2008 and 2009–2011.

**Results:**

In all the three periods analyzed, genotype 1b is predominant (51.8% in 2006–08, 48.3% in 2009–11 and 54.4% in 2012–14) while genotype 2 showed an increase in prevalence (27.9% in 2006–08, 31.7% in 2009–11 and 35.2% in 2012–14) and genotypes 3a and 1a a decrease during the same period (6.8% in 2006–08, 4.7% in 2009–11 and 3.2% in 2012–14 and 7.9% in 2006–08, 4.7% in 2009–11 and 2.6% in 2012–14, respectively). Subtype 1b seems to be equally distributed between males and females (52.7% vs 56.6%) and the prevalence in the age range 31–40 years is significantly higher in the 2012–14 period than in both previous periods (53.8% vs. 16.6% in 2009–11, p< 0.001 and 13.4% in 2006–08, p < 0.001).

**Conclusions:**

Genotype 1b is still the most prevalent, even if shows a significantly increase in the under 40 years old population. Instead, genotype 3a seems to have a moderate increase among young people. Overall, the alarming finding is the “returning” role of the iatrogenic transmission as risk factor for the diffusion of Hepatitis C infection.

## Introduction

Hepatitis C virus (HCV) is one of the most important Flaviviridae infection diffused worldwide and related, as widely described for Hepatitis B virus (HBV) and Hepatitis Delta virus (HDV), to chronic liver disease and Hepatocellular carcinoma development [1–6]. Globally, every year about 3–4 million people are newly infected, and over 350,000 patients die for HCV-related disorders [2].

We recently reported that total global HCV prevalence, increased from 2.3 to 2.8% between 1990 and 2005 [7–9], is currently estimated at 2.5% (177.5 million of HCV infected adults), ranging from 2.9% in Africa and 1.3% in Americas, with a global viraemic rate of 67% (118.9 million of HCV RNA positive cases), varying from 64.4% in Asia to 74.8% in Australasia [10]. These data, if compared to a previous analysis [8], seems to indicate a moderate decrease of global anti-HCV prevalence (-0.3%), especially observed in Western Europe (-1.5%), Southern Africa (-1.2%) and Australasia (-0.9%). Similar data were reported from Europe where the estimated prevalence and number of HCV infected patients, if compared to a similar study about the period 1990–2005 [8], has reduced from 2.6% to 1.7% and from 19 to 13 million [11]. If compared to what previously reported [12–16], a similar decrease it has been observed also in Italy where our data show that currently anti-HCV prevalence is estimated at 3.0% [17], although no updated information are available from Southern Italy, one of the major HCV endemic area in the whole Europe, where we previously described a higher prevalence (about 8.0%) [18–19].

Even though these data appear to indicate an overall decrease of Hepatitis C infection, particularly in resource-rich countries, recent finding has estimated that, in absence of a global strategy through the new DAA therapies, the mortality due to HCV is predicted to increase in the next years [20]. So, although many data suggest that HCV infection could be eliminated in the next twenty years by enhancement in development of strategies to cure the patients already infected and ovoid new infections [21,22], more studies are needed to understand deeply the HCV epidemiology and then develop strategies to eradicate HCV.

Until now, HCV genotypes are divided into seven recognized subtypes on the basis of sequence of the viral genome, that may differ from each other by 30%-35% of nucleotide sites and into over 67 subtypes (a, b, c, etc.), differing at <15% of nucleotide sites [23,24] which have different geographical distribution [8,10]. Some of them, specifically 1a, 1b, 2a, and 3a, called “endemic subtypes”, probably spread in the 70’s, before HCV sequencing and through transfusion, blood products and people who insert drugs (PWID), are widely distributed worldwide and account for a great proportion of the totality of HCV cases, especially in resource-rich countries [23–26]. Otherwise, the so called “endemic strains”[7–10], are comparatively infrequent and have been limited for long time in specific regions, as West Africa, Southern Asia, Central Africa and South Eastern Asia [27–28]. At present, genotype 7 infection has been reported from 4 patients originating from the Democratic Republic of Congo [29].

The heterogeneity among HCV genotypes in geographic distributions reflects the differences in epidemiology, like modes of transmission and ethnic variability in different countries. Globally, HCV genotype 1 is the most prevalent worldwide (49.1%), followed by genotype 3 (17.9%), 4 (16.8%) and 2 (11.0%), while genotypes 5 and 6 are responsible for the remaining < 5% [8]. HCV genotypes 1 and 2 are distributed globally, while genotypes 4, 5, 6 and 7 are common to more specific geographical areas [30].

Although genotypes 1, 2 and 3 are prevalent worldwide, their relative prevalence varies greatly from one geographic area to another. HCV subtypes 1a and 1b are the most common in Northern America, Europe and Japan [10,31–32] while genotype 2 in West Africa and in South America, probably caused by population movements during the trans-Atlantic slave trade in the 1700 [10,32] and in some regions of Italy [17–19, 33]. Subtype 3a, which is very common among intravenous drug abusers, is common mainly in Europe, USA and South East Asia while genotype 4 prevails in North Africa and Middle East and genotypes 5 and 6 are endemic, respectively, in South Africa and in South China [10, 31–32, 34–35].

Data recently published showed that in the overall Italian population the predominant genotypes is 1 (64,7%), followed by 2 (26.0%), while genotypes 3 and 4 are both estimated under 4% [31], although these data seems to change considerably in the PWID population where the genotype 3 is the most prevalent (41,3%) followed by subtypes 1a (23,1%) and 1b (20,6%) [36–37]. It is important to consider, anyway, that these data were extrapolated from studies restricted to limited populations.

Focus of our paper was to provide updated finding on the changing epidemiology of Hepatitis C infection and on the prevalence of the various HCV genotypes in Southern Italy, studying three different groups of HCV positive patients in the periods 2006–2008, 2009–2011 and 2012–2014.

## Materials and methods

### Study population and sample collection

We enrolled 63055 outpatients all coming from different cities of the Southern Italian Regions (Campania, 65.0%; Molise 5.0%; Puglia 5.0%; Basilicata 12.0%; Calabria 13.0%), consecutively recruited only at Istituto Nazionale Tumori, IRCCS “Fondazione G. Pascale”, Naples, from January 2006 to December 2014, related to three-year periods 2006–2008, 2009–2011 and 2012–2014. No data were available from Sicily. Since not all anti-HCV positive sera were available, HCV RNA determination was carried out only on the available sera. Features of the selected patients for each three-year period are indicated below:

a) 2006–2008

Out of 16275 subjects initially recruited, 1302 (8.0%) were anti-HCV positive. HCV-RNA test was performed in 652 of them (50.1%) with a viraemic rate of 54,0% (352/652). Among these 352 patients, 176 (89 males and 87 females, both with a mean age of 53.5 years, range 25–82) were included in the study and genotyped.

b) 2009–2011

Out of 21108 subjects initially recruited, 1562 (7.4%) were anti-HCV positive. HCV-RNA test was performed in 798 of them (51.1%) with a viraemic rate of 55,0% (439/798). Among these 439 patients, 255 (118 males and 137 females, with an average age of 50.5 years for males, range 18–83, and 47.5 years for females, range 18–77) were included in the study and genotyped.

c) 2012–2014

Out of 25672 subjects initially recruited, 1643 (6.4%) were anti-HCV positive. HCV-RNA test was performed in 871 of them (53.0%) with a viraemic rate of 61.4% (535/871). Among these 535 patients, 349 of them (197 males and 152 females, with an average age of 51.5 years for males, range 18–85, and 50.0 years for females, range 18–82), were included in the study and genotyped.

Exclusion criteria for the study were: HCV-RNA negative samples or, as previously specified, insufficient or unavailable serum for HCV-RNA determination or for genotyping. No significant differences of gender or age were found between the analyzed subjects and the excluded patients.

Information about risk factors for Hepatitis C were available for all the included patients. In case multiple risk factors the one most likely to associated with an increase risk of disease was assigned. Anyway, it is interesting to note that, while dental therapy is an isolated risk factor (over 95% of the selected patients), we detected a very close correlation between drug abuse and tattoos (56%). Instead, surgery, is generally equally associated to all the other risk factors. It is important to highlight, anyway, that even counting each risk factor separately, analysis of the results doesn’t change significantly.

All patients enrolled were asymptomatic, negative for anti-HIV, HBsAg and anti-HDV and had no clinical symptoms or biochemical markers of other chronic liver disease (autoimmune disorder, non-alcoholic fatty liver disease, hemocromatosis, etc.). No patient admitted alcohol abuse defined as the consumption above 30g pure alcohol per day for females and 40g / day for males in the last 6 months.

No differences in pattern of sampling or testing used in the three considered periods. The Ethic Committee of Istituto Nazionale Tumori—Fondazione “G. Pascale*”*granted approval for the study that was conducted according the principles of ICG-GCP and declaration of Helsinki. Prior written informed consent was obtained from each patients and all data was de-identified using data collection.

### Serology and molecular analysis

We detected the positivity of anti-HCV antibodies in all plasma samples by Vitros ECi test (Ortho Clinical Diagnostics), used according to the manufacturers’ instructions.

The Ortho-Clinical Vitros ECi test is an immunoassay system in which a positive antibody-antigen reaction create a light signal that is directly proportional to the amount of antibody present. Results are reported as signal to cut off and values≥1.00 are considered reactive for HCV antibodies. The test is highly specific and sensitive (99.97% and 100%respectively).

Only repeatedly anti-HCV positive samples were subsequently tested for the detection and quantification of viral genome by Polymerase Chain Reaction (PCR) in Real Time by means of COBAS Ampliprep/COBAS Taqman HCV (Roche Diagnostics System Inc.). Linear range of assay was 1.50 x 10^1^ to 6.90 10^7^ IU/ml, with the accuracy acceptance criterion of +/- 0.3 log^10^. The test had a specificity of 100% and its limit of detection (LOD) was 15 IU/ml.

HCV genotyping was assessed using the Versant HCV Genotype Assay 2.0 LiPA, a fully automated system (Siemens Healthcare Diagnostics). Viral genome is first amplified and then viral fragments are hybridized by means of genotype-specific probes bounded onto nitrocellulose strips. The test have a specificity and sensitivity of 96% and 99.4% respectively, and its LOD is 15 IU/ml. No mixed infections were described.

### Statistical analysis

All statistical analysis was done through SPSS software, version 17. The χ^2^ tests was used to analysed the frequency tables and the correlation between the categorical variables was assessed using the Pearson correlation. Student’s *t* test was used to analyse differences in the mean ages in the genotypes and subtypes. In all tests, p*-*values< 0.05 indicates a strong evidence for statistical significance.

Multiple logistic regression analysis was used to estimate the odds ratio of genotype prevalence in relation to gender and age in the analysed periods.

## Results

**Fig 1:**
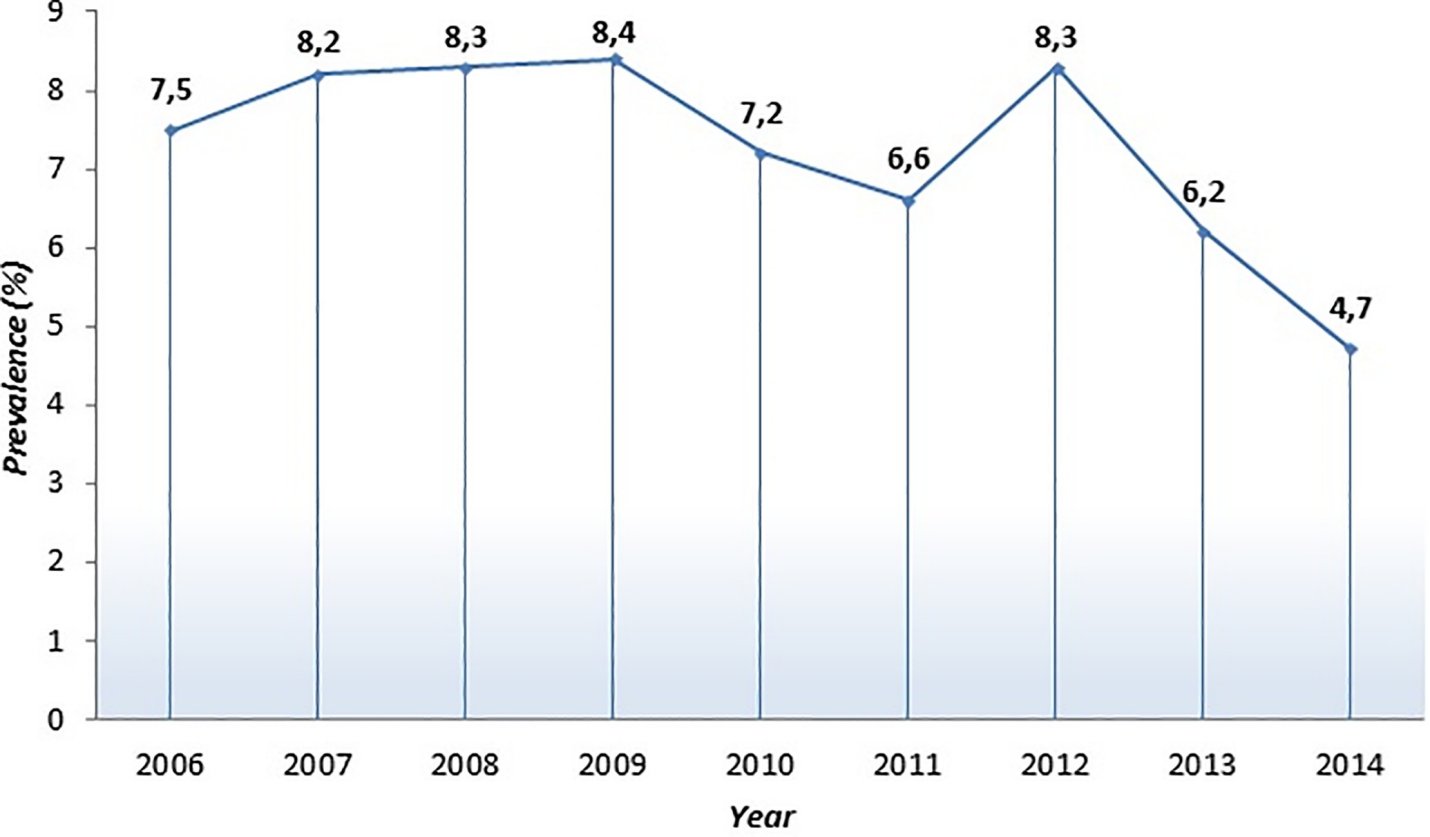
Prevalence of HCV-Ab positive patients from 2006 to 2014.

Prevalence of anti-HCV during the nine-year of analysis (from 2006 to 2014) is reported in Fig 1 and shows a marked decrease (-2.8%, p<0,00001), while the viraemic rate in the same period exhibits an evident increase (+ 13.0%, p<0.005).

**Fig 2:**
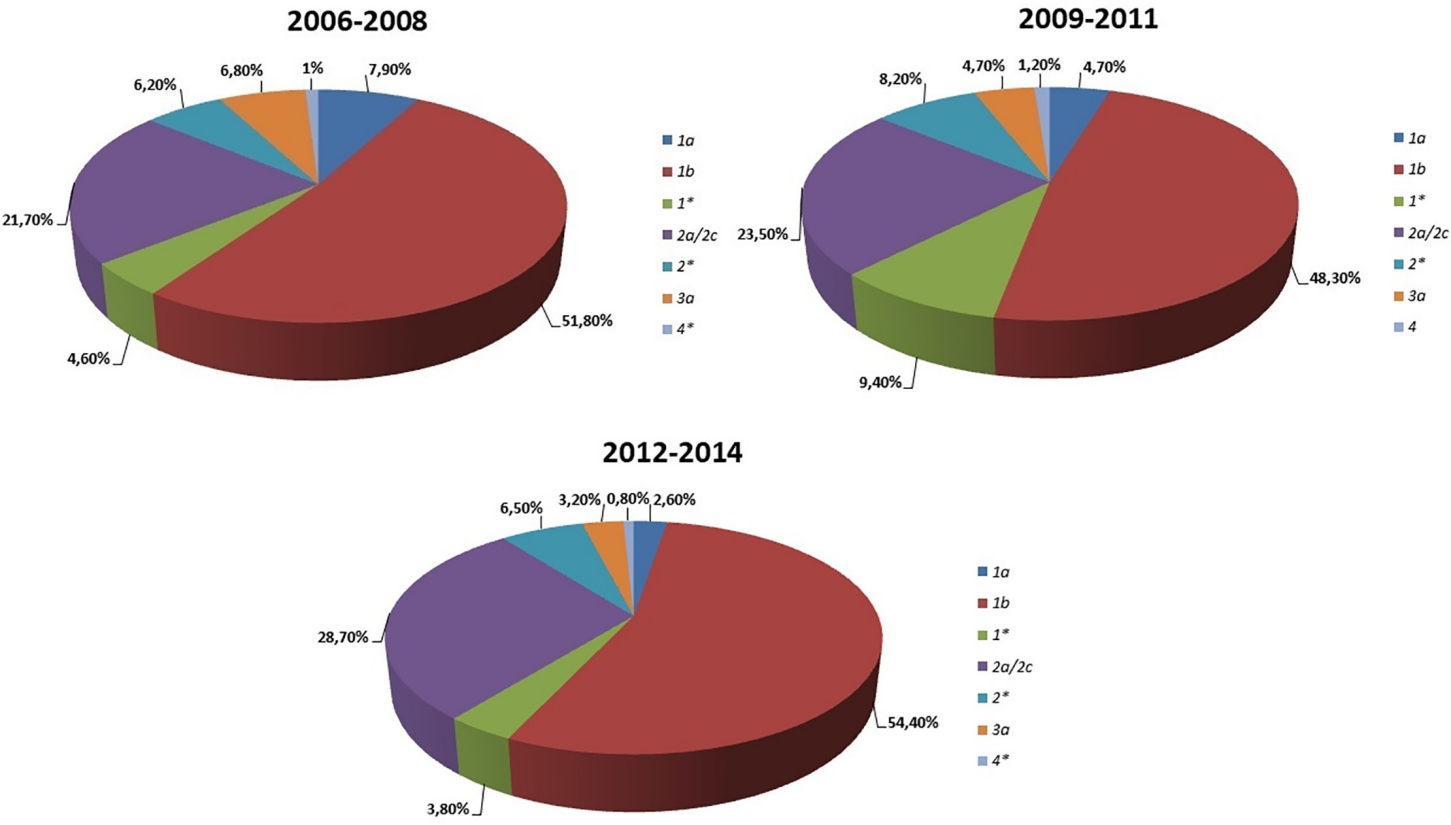
Distribution of HCV genotypes in three–year periods 2006–2008, 2009–2011 and 2012–2014.

**Fig 3:**
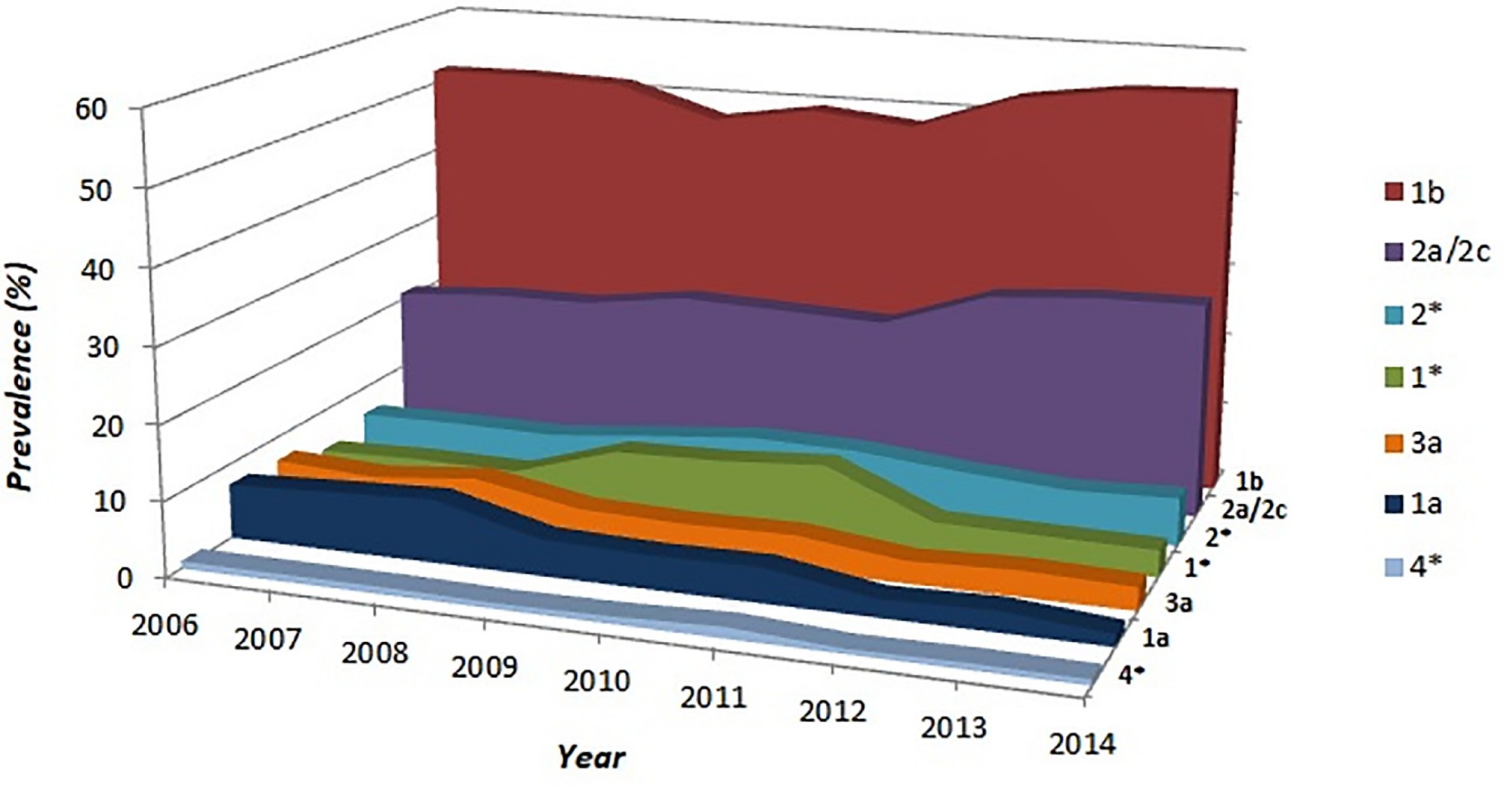
Distribution of HCV genotypes from 2006 to 2014.

The prevalence of the genotypes among the patients during the three analysed periods is illustrated in Fig 2. All subtype 3 genotype were subtype 3a, and no case of genotypes 5 and 6 were identified. Genotype 1b is the most frequent in all the three periods, even if its prevalence decreased from 2006–2008 to 2009–2011 from 51.8 to 48.3% and on the contrary increased from 2012 to 2014 (approximately + 6.0%) (Fig 3). At same time we observed a similar increase in the rate of genotype 2 (including subtype 2a/2c and non-subtypable genotype 2) from 2012 to 2014 (approximately + 7.0%)(Figs 2 and 3).

In this paper, subtypes 1* and 2* (not further subclassified) constitute the 3rd and the 4th largest groups, respectively, in the 3-year period 2009–2011 (9.4 and 8.2%) and 2012–2014 (3.8% and 6.5%). During the nine years studied subtype 3a gradually decreased from 6.8 to 3.2%, as genotype 1a from 7.9 to 2.6% (p<0.05). It is important to point that the distribution of subtypes 1a and 1b during the nine years could significantly change since the exact classification of not-further-subtypable genotype 1* is not completely clear. Genotype 4 during the analysed period proved to be the most constant with an overall prevalence of 1.0% (Fig 3).

Gender-related differences observed among the subtypes are reported in Table 1. Genotype 1b in the past more frequently observed among females than males (39.3% of 89 males vs. 64.3% of 87 females in 2006–2008, p<0.001; 41.5% of 118 males vs. 54.0% of 137 females in 2009–2011, p<0.05), it seems not show any significant differences (52.7% of 197 males vs 56.6% of 152 females) in the three-year period 2012–2014.

**Table 1:** Gender distribution of HCV subtypes in Southern Italy in the three-year periods 2006–2008, 2009–2011 and 2012–2014.

Genotype	2006–2008				2009–2011				2012–2014			
	Male		Female		Male		Female		Male		Female	
	n.	%	n.	%	n.	%	n.	%	n.	%	n.	%
**1**	50	56.2	63	72.4	67	56.8	92	67.1	120	60.9	92	60.5
**1a**	7	7.9	7	8.1	6	5.1	6	4.4	8	**4.1**^**F**^	1	**0.7**^**F**^
**1b**	35	**39.3**^**A**^	56	**64.3**^**A**^	49	**41.5** ^**B**^	74	**54.0** ^**B**^	104	52.7	86	56.6
1*	8	9.0	0	0.0	12	10.2	12	8.7	8	4.1	5	3.2
**2**	27	30.3	22	25.3	39	33.0	42	30.7	67	34.0	56	36.8
**2a/2c**	18	20.2	20	23.0	27	22.8	33	24.1	53	26.9	47	30.9
2*	9	10.1	2	2.3	12	10.2	9	6.6	14	7.1	9	5.9
**3a**	12	**13.5**^**C**^	0	**0.0** ^**C**^	12	**10.2** ^**D**^	0	**0.0** ^**D**^	8	**4.1**^**E**^	3	**2.0**^**E**^
4*	0	0.0	2	2.3	0	0.0	3	2.2	2	1.0	1	0.7
**Total**	**89**	**100.0**	**87**	**100.0**	**118**	**100.0**	**137**	**100.0**	**197**	**100.0**	**152**	**100.0**

A = p< 0.001; B = p< 0.05; C = p<0.0005; D = p<0.0005; E = p<0.05; F = p <0.001

* Other cases not further sub classified.

On the contrary, genotype 3a is more prevalent in males than in females patients (13.5 vs. 0% in 2006–2008, p*<*0.0005; 10.2 vs. 0% in 2009–2011, p<0.0005; 4.1 vs 2.0% in 2012–2014, p*<*0.05), possibly due to a higher prevalence of PWID among men, even if the male/female ratio seems to be markedly decreased from 2006 to 2014.

Genotype 1a previously equally distributed between females and males (8.1% vs 7.9% in 2006–2008, 4.4% vs 5.1% in 2009–2011), appears to have now a statistically significant increase in males compared to females (4.1% vs. 0.7% in 2012–2014, p <0.001). Genotype 4, instead, that in the past showed an exclusive prevalence among females (2.3% in 2006–2008, 2.2% in 2009–2011), appears now to be equally distributed among males and females (1.0% vs 0.7% in 2012–2014), even these differences were not found to be statistically significant. Analyzing data as a continuum from 2006 to 2014 the general meaning does not change.

**Fig 4:**
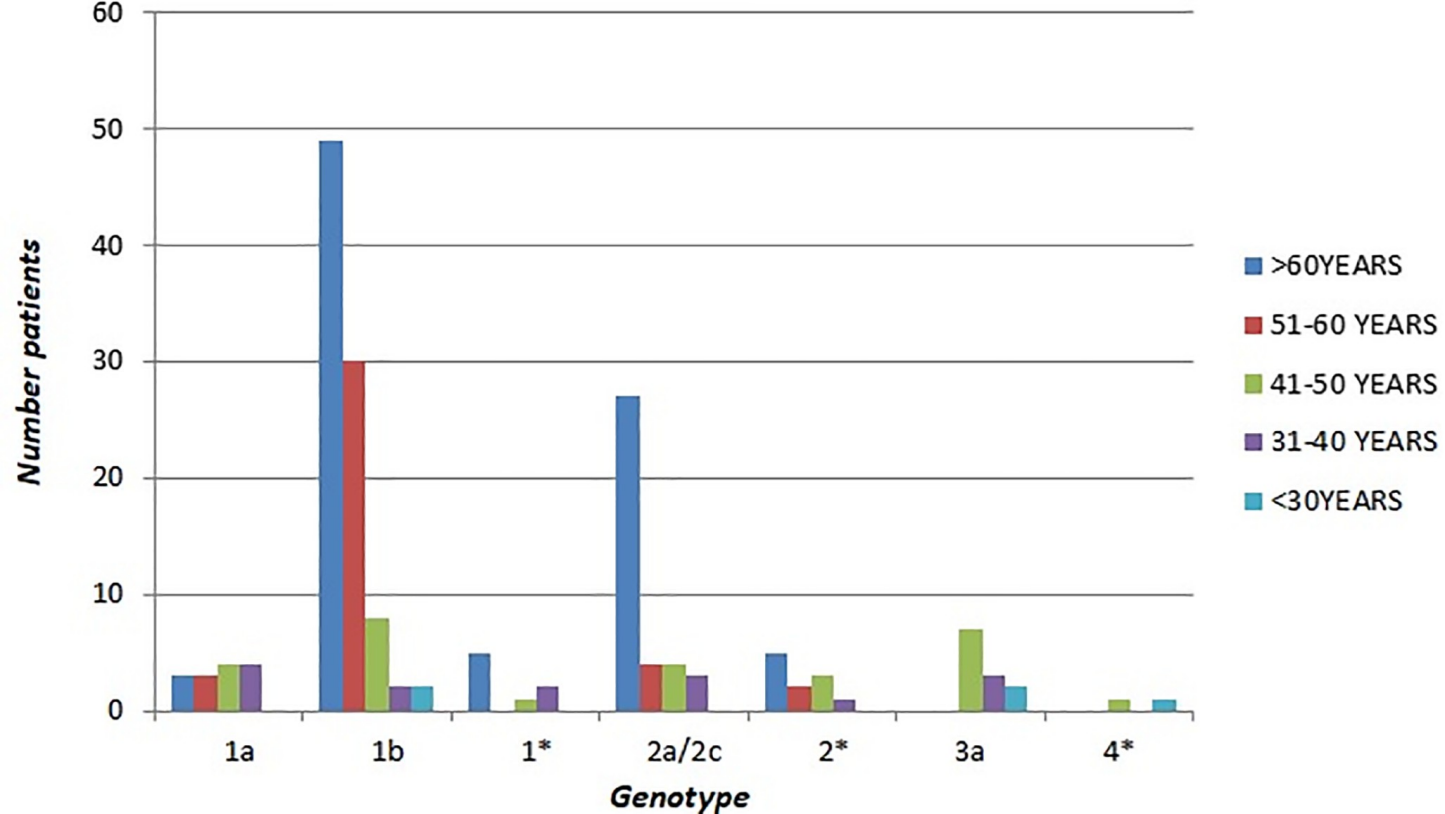
Age distribution of HCV genotypes in 2006–2008.

**Fig 5:**
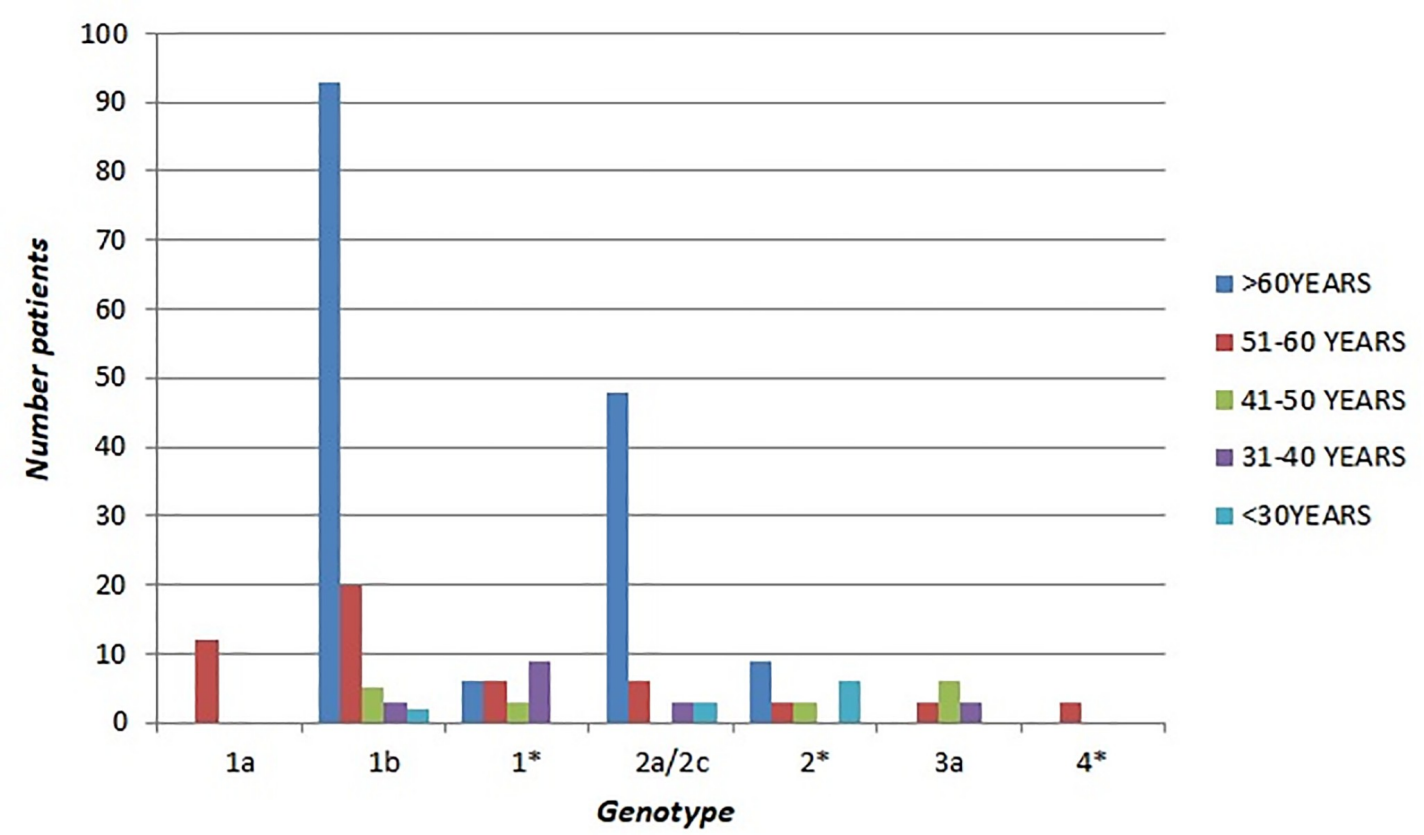
Age distribution of HCV genotypes in 2009–2011.

**Fig 6:**
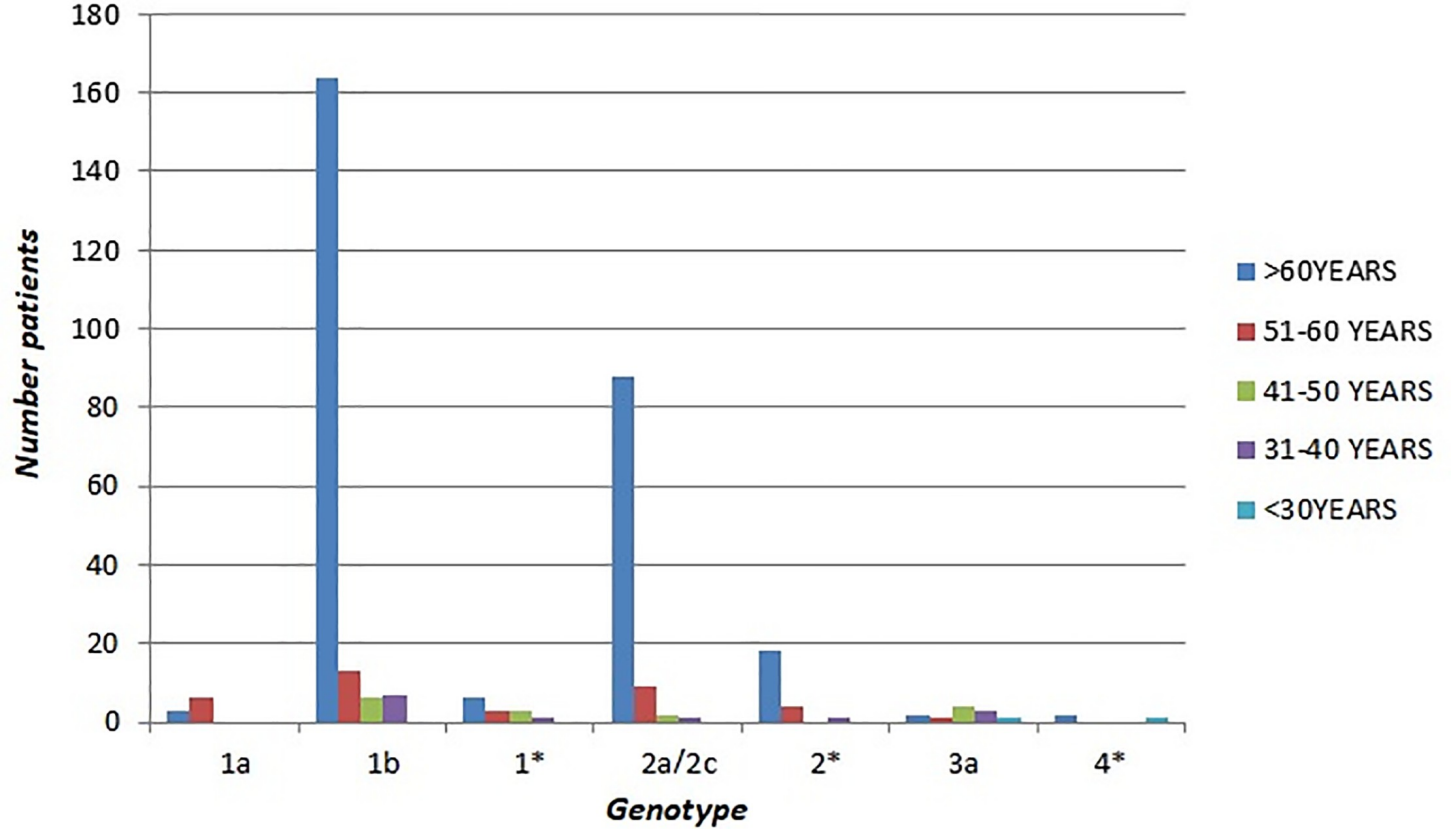
Age distribution of HCV genotypes in 2012–2014.

To further compare what kind of HCV genotypes prevails in accordance with age patients, these were grouping into 5 age groups, as shown in Tables 2, 3 and 4. The results showed a significant variation in the distribution according to age in the three periods. Genotype 1b was the most common in the over-50 age patients compared to 50 and under: in fact, in 2006–2008 period, 79 of 128 subjects (61.7%) older than 50 had genotype 1b, a higher percentage than that observed among the 48 patients of less than 51 years of age (12/48, 25%, p<0.0001); in 2009–2011, of 209 over 50 year-olds, 113 (54.1%) had genotype 1b, vs. 10 of the 46 (21.7%) who were younger than 50 years of age (p< 0.0001). Similar data were also found in the 2012–2014 period where among the 319 over 50 years-old patients genotype 1b was found in 177 of them (55.5%) vs. 13 of the 30 (43.3%) who were younger than 50 years. It is interesting to point that in the 31–40 age group genotype 1b was significantly more common in the period 2012–2014 than in both the previous periods (53.8% in 2012–2014 vs. 16.6% in 2009–2011, p <0.001) and (53.8% in 2012–2014 vs. 13.4% in 2006–2008, p <0.001) (Figs 4–6).

**Table 2:** Age distribution of HCV genotypes in 2006–2008.

Genotype	> 60 years		51–60 years		41–50 years		31–40 years		< 30 years		Total
	n.	%	n.	%	n.	%	n.	%	n.	%	
**1**	57	64.1	33	84.6	13	46.4	8	53.4	2	40.0	113
** 1a**	3	3.4	3	7.7	4	14.3	4	26.6	0	0.0	14
** 1b**	49	55.1	30	76.9	8	28.5	2	13.4	2	40.0	91
1*	5	5.6	0	0.0	1	3.6	2	13.4	0	0.0	8
**2**	32	35.9	6	15.4	7	25.0	4	26.6	0	0.0	49
** 2a/2c**	27	30.3	4	10.3	4	14.3	3	20.0	0	0.0	38
2*	5	5.6	2	5.1	3	10.7	1	6.6	0	0.0	11
**3a**	0	0.0	0	0.0	7	25.0	3	20.0	2	40.0	12
4*	0	0.0	0	0.0	1	3.6	0	0.0	1	20.0	2
**Total**	**89**	**100.0**	**39**	**100.0**	**28**	**100.0**	**15**	**100.0**	**5**	**100.0**	**176**

* Other cases not further subclassified.

**Table 3:** Age distribution of HCV genotypes in 2009–2011.

Genotype	> 60 years		51–60 years		41–50 years		31–40 years		< 30 years		Total
	n.	%	n.	%	n.	%	n.	%	n.	%	
**1**	99	63.4	38	71.6	8	47.0	12	66.8	2	18.2	159
** 1a**	0	0.0	12	22.6	0	0.0	0	0.0	0	0.0	12
** 1b**	93	59.6	20	37.7	5	29.4	3	16.6	2	18.2	123
1*	6	3.8	6	11.3	3	17.6	9	50.2	0	0.0	24
**2**	57	36.6	9	17.0	3	17.6	3	16.6	9	81.8	81
** 2a/2c**	48	30.8	6	11.3	0	0.0	3	16.6	3	27.3	60
2*	9	5.8	3	5.7	3	17.6	0	0.0	6	54.5	21
**3a**	0	0.0	3	5.7	6	35.4	3	16.6	0	0.0	12
4*	0	0.0	3	5.7	0	0.0	0	0.0	0	0.0	3
**Total**	**156**	**100.0**	**53**	**100.0**	**17**	**100.0**	**18**	**100.0**	**11**	**100.0**	**255**

* Other cases not further subclassified.

**Table 4:** Age distribution of HCV genotypes in 2012–2014.

Genotype	> 60 years		51–60 years		41–50 years		31–40 years		< 30 years		Total
	n.	%	n.	%	n.	%	n.	%	n.	%	
**1**	173	61.1	22	61.1	9	60.0	8	61.5	0	0.0	212
** 1a**	3	1.1	6	16.7	0	0.0	0	0.0	0	0.0	9
** 1b**	164	57.9	13	36.1	6	40.0	7	53.8	0	0.0	190
1*	6	2.1	3	8.3	3	20.0	1	7.7	0	0.0	13
**2**	106	37.5	13	36.1	2	13.3	2	15.4	0	0.0	123
** 2a/2c**	88	31.1	9	25.0	2	13.3	1	7.7	0	0.0	100
2*	18	6.4	4	11.1	0	0.0	1	7.7	0	0.0	23
**3a**	2	0.7	1	2.8	4	26.7	3	23.1	1	50.0	11
4*	2	0.7	0	0.0	0	0.0	0	0.0	1	50.0	3
**Total**	**283**	**100.0**	**36**	**100.0**	**15**	**100.0**	**13**	**100.0**	**2**	**100.0**	**349**

* Other cases not further subclassified.

Regarding genotype 1a, we found that its prevalence among the 51–60 years old patients was higher in the three-year periods 2012–2014 and 2009–2011 although a decrease over time (22.6% in 2009–2011 vs 16.7% in 2012–2014) (Figs 4–6).

Instead, the prevalence of genotype 2 (including subtype 2a/2c and non-subtypable genotype 2) showed no difference among age groups throughout the analysed periods with the exception for the 51–60 age group that showed a statistically significant increase in 2012–2014 when compared to both the previous periods (15.4% in 2006–2008 vs. 36.1% in 2012–2014, p<0.001) and (17.0% in 2009–2011 vs. 36.1% in 2012–2014, p<0.001) (Figs 4–6). As regards genotype 3a, its prevalence was higher in younger patients (25% of the 48 patients ≤50 years old vs. none of 128 ≥ 51 years old, p<0.0001 in the period 2006–2008; 19.6% of the 46 patients ≤51 years old vs. 1.4% of the 209 ≥ 50 years old in the period 2009–2011 (p<0.0001); 26.6% of the 30 ≥ 50 years old in the period 2012–2014 vs. 0.9% of the 319 older than 50 years (p<0.0001) (Figs 4–6).

Analysing risk factors more commonly related to the acquisition of HCV infection (Table 5), we observed that dental therapies, previously the "emerging" risk factor for the acquisition of HCV infection, show a significant decrease in the period 2012–2014 (-15.8%, from 40.7% in 2009–2011 to 24.9% in 2012–2014, p<0.05), while infections related to surgery show a significant increase (+ 25.1%; from 11.8% in 2009–2011 to 36.9% in 2012–2014, p<0.001). It is important to highlight that this risk factor gradually increase in the last period from 2012 to 2014. (data not shown). Conversely, risk related to piercing and/or tattoos and blood transfusions both show a decrease (3.4% in 2006–2008 vs 0.6% in 2012–2014 p<0.05 and 13.6% in 2006–2008 vs. 8.0% in 2012–2014 p<0.05, respectively). No significant changes were observed over time for drug addiction as risk factor (7.4% in 2006–2008, 6.3% in 2009–2011 and 9.0% in 2012–2014).

**Table 5:** Route of transmission for Hepatitis C virus infection in Southern Italy from 2006 to 2014.

Route of transmission^§^	2006–2009		2009–2011		2012–2014		Total	
	n.	%	n.	%	n.	%	n.	%
**Unknown**	45	25.6	71	27.8	64	18.3	180	23.0
**Dental Therapy**	52	29.6	104	40.7^*^	87	24.9^*^	243	31.1
**Surgery**	27	15.3	30	11.8^**^	129	36.9^**^	186	23.8
**Blood transfusion**	24	**13.6** ^***^	16	6.3	28	**8.0** ^***^	68	8.8
**Intravenous drug use**	13	7.4	16	6.3	31	9.0	60	7.7
**Piercing/tattoo**	6	**3.4** ^****^	4	1.6	2	**0.6** ^****^	12	1.6
**Needle stick injuries**	9	5.1	14	5.5	8	2.3	31	4.0
**Total**	**176**	**100.0**	**255**	**100.0**	**349**	**100.0**	**780**	**100.0**

* = p <0.05 (2009–2012 vs 2012/2014)

** = p <0.001 (2009–2012 vs 2012/2014)

*** = p <0.05 (2006–2009 vs 2012/2014)

**** = p <0.05 (2006–2009 vs 2012/2014).

§ In case of more than one risk factor the one most likely to have caused infection was assigned

Multiple logistic regression analysis was used to assess the meaning of HCV genotypes prevalence during the nine-year periods. The different distribution of genotypes was independently associated with gender and age. Our analysis showed that all the differences in HCV genotype distribution were significant and not influenced by the variability of gender or age group.

## Discussion

HCV genotypes are widely distributed throughout the world and their distribution varies mostly according to the geographical region [10]. It is well known that HCV subtypes play a crucial role in the therapeutic approach, since the severity and the prognosis of the disease may vary greatly according to the different genotype [3, 38–39].

Although findings recently published show a global decrease of HCV infection, particularly in resource-rich countries [10], likely due to the introduction of the new antiviral therapies, some prospective modelization studies suggest that HCV-related mortality will increase in the next years [20–21] since the use of classic therapeutic approach "genotype-dependent" is still largely common, especially in resource-poor countries. This implies that a deeply knowledge of HCV epidemiology is still necessary to create strategies needed to eradicate HCV pandemic.

As previously globally described [10], also data obtained in this regional study suggest a decrease in the prevalence of HCV in Southern Italy from 2006–2008 to 2012–2014 (-1.6%) and a contemporary increase of the viraemic rate (+7.4%) for sure related to the aging of infected population.

Our data seems to suggest some interesting changes in the epidemiology of HCV genotypes in Southern Italy over the nine years. Genotype 1b is historically the most prevalent, both in Southern Italy and throughout the whole Italy [18,40–41] and still remains so (54.4%), followed by genotype 2a/2c (28.7%) that however shows a marked increase from 2006–2008 to 2012–2014 (+7.0%). It is likely that the migration fluxes from Balkan area to Italy may have caused an increase prevalence of genotype 2 in the Southern Italy [17]. As we recently described, comparing our data with those collected by The Polaris Observatory [42], G2, typical of Albania [43], seems to increase its prevalence in the last years only in Italy (+12.0%) without any significant change in the other Southern Europe countries [18–19,33,44–45]. Although some hypothesis suggest that G2 was probably introduced in Italy as a consequence of Albanian campaign during Second World War [33], it is more likely that the migration fluxes from Albania to Italy in the 90s may have increased its prevalence in the Southern Italy [17].

On the other hand, genotypes 1a and 3a, considerably less common in our area (2.6 and 3.2%, respectively), compared to data from other regions of Italy [41] exhibit a drastic decrease during the nine studied years (-5.3% and -3.6%, respectively), even though these data could be modified by the “not further classified” genotype 1* whose classification is still unclear. Regarding genotype 4, recently documented in some Mediterranean countries, such as Albania, Spain and Greece [46–48] and probably related to migratory flows from Middle Eastern countries and Northern Africa, its presence do not seems to be relevant in our area, at least until 2014, showing a stable prevalence in the three periods studied.

The gender distribution of HCV genotypes shows, as previously described [8–11], a marked prevalence of genotypes 1a and 3a among males (4.1% vs. 0.7% and 4.1 vs 2.0%, respectively), probably due to a higher prevalence of PWID among men, even if, especially for genotype 3a, the male/female ratio seems to be decreased from 2006 to 2014. Subtype 1a, before equally distributed between females and males (8.1% vs. 7.9% in 2006–2008, 4.4% vs. 5.1% in 2009–2011) appears to have now a statistically significant increase in males. Conversely, genotype 1b in the past more frequently observed among females (64.3% vs. 39.3% in 2006–2008 and 54.0% vs. 41.5% in 2009–2011) seems not show any significant differences now between females and males (56.6% vs. 52.7% in 2012–2014).

Considering genotypes distribution related to age, genotype 1b, as widely reported in literature [49–55], is considerably more frequently in the older patients, especially over 50 years, although it is interesting to highlight its increasing in the 31–40 age group in 2012–2014 period if compared to the two previous periods (+37.2% respect to 2009–2011 and + 40.4% respect to 2006–2008). Since this genotype is historically correlated to transfusion-related transmission, this new peak in younger patients might be related to an emerging new transmission route. Instead, evaluating age distribution of genotype 2 (including subtype 2a/2c and non-subtypable genotype 2) it is evident its statistically significant increase in patients over 50 years in the three-year period 2012–2014 when compared to both the previous periods (+7.2% and +7.6%, respectively). This finding seems to confirm the reduced risk of transfusion-associated transmission in the years and in the meantime highlights the increasing role of no-age related risk factors in diffusion of HCV infection. On the contrary, subtype 3a is greatly present in younger patients showing a moderate increase in the age group < 30 years old (+10% from 2006–2008 to 2012–2014), even if these data must be confirmed for the reduced number of patients belonging to genotype 3 examined.

Data obtained by analysis of risk factors in the three studied periods show clearly that blood transfusions contribute minimally on the onset of new infections because of the more and more safer screening procedures globally adopted. Dental therapy, described in the first decade of new millennium as one of the major risk factor for the acquisition of HCV infection in Southern Italy [18], on the contrary, seems to have now a secondary role, as also documented by the increased prevalence of genotype 2 in the aged population. While PWID seems constantly to play a minor role in introducing new HCV infections in Southern Italian population, the increased incidence of surgery practices as risk factor and the increasing prevalence of genotype 1b in the 31–40 age group suggest a possible new role of the iatrogenic infections in the diffusion of HCV infection in Southern Italy. The analysis of risk factors, in fact, shows a marked increase of infections caused by iatrogenic factors from 2011to 2014 of about 25%.

Comparative analysis of the three 3-year period 2006–2008, 2009–2011 and 2012–2014 reveals an interesting trend in the genotypes distribution in Southern Italy. Genotype 1b is the most dominant among elderly patients, probably for a cohort effect. It was supposed that these patients, infected several years ago when genotype 1b was probably the only one existing in the area with an high incidence, have maintained their old infection, among a pool of younger carriers initially infected by genotype 1a and then by genotype 3a. In fact, a high prevalence of genotypes 3a in under-40-year-old subjects and of genotype 1a in 30–50 years old patients was observed in the period 2006–2008. This was not confirmed in the three-year periods 2009–2011 and 2012–2014 in which there was a reduction in the genotype 3a frequency in subjects under 30 years of age and in the meantime a moving of genotype 1a infected patients toward old age groups. The decreasing role of dental therapy as risk factor might explain the increasing of genotype 2 in the elder population.

Multivariate analysis indicated that the different distribution of genotypes 3a and 1b during the analysed periods was independently associated with any variability in gender and/or age group.

By the analysis of our data it can be speculated that genotype 1b was the native “resident” one in Southern Italy, widespread in the seventies, especially through iatrogenic infections and the use of non-sterile syringes. The introduction of HCV screening programmes in the last decades of twentieth century have drastically reduced the infection risk, thereby generating the cohort effect we now describe. Genotype 2, the second “resident” genotype in Italy, whose circulation has been initially implemented through unsafe dental practices and then for immigration fluxes from Balkan area, especially from Albania, at the end of 90’s, has become recently more and more common in the elder population. This effect could have been caused by the more and more safer dental procedures in our area as demonstrated by the reduced percentage of dental-related infections observed in the 2012–2014 period. Regarding intravenous drug abuse might have led to two different waves of HCV infection especially among young population, introducing before genotype 1a (now more common in aged patients) and then 3a [30].

In conclusion, the epidemiological framework of Hepatitis C infection in Southern Italy, particularly interesting for the high prevalence of this virus in the general population, seems to highlight the "returning" role of the iatrogenic transmission as risk factor for the diffusion of HCV infection. Furthermore, the small increase of genotype 3a among young people should be more investigated, with a support of a phylogenetic analysis.

At support of our hypothesis, some studies report small HCV outbreaks in Europe due to breaches in standards of health and safety practices among health-care workers [56]. Indeed, an interesting case–control study highlighted some unconventional routes of diffusion of Hepatitis C infection such as digestive endoscopy, beauty treatments and professional pedicure/manicure [57]. This suggest not only a necessary evaluation of the safety practices in surgery, but the fundamental importance of not lowering the safety levels, especially among all health-care professionals.

## Abbreviations:

HCV: Hepatitis C Virus
HCC: Hepatocellular Carcinoma
RNA: Ribonucleic Acid
RT-PCR: Reverse transcription polymerase chain reaction
IU/mL: International Units per Milliliter
LiPA: Line Probe Assay
SPSS: Software package used for statistical analysis
HBV: Hepatitis B virus
