# Correction: Nine-year distribution pattern of hepatitis C virus (HCV) genotypes in Southern Italy

**DOI:** 10.1371/journal.pone.0215559

**Published:** 2019-04-12

**Authors:** Arnolfo Petruzziello, Rocco Sabatino, Giovanna Loquercio, Annunziata Guzzo, Lucia Di Capua, Francesco Labonia, Anna Cozzolino, Rosa Azzaro, Gerardo Botti

The affiliation for author Gerardo Botti is incorrect. The correct affiliation is: Scientific Director, Istituto Nazionale Tumori, Fondazione “G. Pascale”, IRCCS Italia, Naples, Italy.
